# Recent Research Progress in the Abrasive Machining and Finishing of Additively Manufactured Metal Parts

**DOI:** 10.3390/ma18061249

**Published:** 2025-03-12

**Authors:** Tesfaye Mengesha Medibew, Dawid Zieliński, Sisay Workineh Agebo, Mariusz Deja

**Affiliations:** 1 Doctoral School, Gdańsk University of Technology, Gabriela Narutowicza Street 11/12, 80-233 Gdansk, Poland; tesfaye.mengesha.medibew@pg.edu.pl (T.M.M.); sisay.workineh.agebo@pg.edu.pl (S.W.A.); 2 Department of Manufacturing and Production Engineering, Institute of Machine and Materials Technology, Faculty of Mechanical Engineering and Ship Technology, Gdańsk University of Technology, Gabriela Narutowicza Street 11/12, 80-233 Gdansk, Poland; mariusz.deja@pg.edu.pl

**Keywords:** additive manufacturing, abrasive machining, surface finish, dimensional accuracy, mechanical properties, nonconventional methods

## Abstract

Additive manufacturing (AM) has revolutionized the production of complex geometrical parts with metals; however, the usual layer-by-layer deposition results in poor surface quality and unpredictable surface integrity. Abrasive machining and finishing techniques play vital roles in counteracting these challenges and qualifying AM parts for practical applications. This review aims to present recent research developments concerning the machining of additively manufactured metal parts via both conventional and nonconventional abrasive machining methods. Conventional methods such as grinding, milling, polishing, honing, and sandblasting have been widely investigated for their ability to enhance the surface finish, dimensional accuracy, and mechanical properties of AM metal components. However, the characteristic features of various AM processes, such as porosity, microstructural features, and residual stresses, can significantly influence the machinability of the produced parts. Nonconventional methods such as abrasive flow machining, electrochemical machining, magnetic abrasive finishing, and vibratory bowl finishing, on the other hand, have shown potential in addressing the difficulties associated with internal machining geometries and hard-to-machine material combinations that are typical for many AM parts. This review also highlights some challenges and future trends in the machining of AM metal parts and emphasizes that further research is required in the direction of combinations of various postprocessing techniques, machinability regarding new alloy compositions, and the integration of AI for process optimization. As the demand for high-precision AM parts grows across various industries, the advancement of abrasive machining and finishing techniques is crucial for driving the wider adoption of AM technologies.

## 1. Introduction

Three-dimensional (3D) printing technology has gained wide popularity over the past few years, owing to its versatility and ability to manufacture complex parts and structures. The ability to fabricate model parts, working systems, and biocompatible parts with complex structures and nanostructures has transformed many industries [1,2,3,4]. These processes have been increasingly utilized over the past two decades to produce complex metal parts, which have significantly increased in size through the melting and layering of raw materials according to digital designs. Additive manufacturing usually requires less postprocessing than traditional manufacturing because it does not require additional external finishing procedures to produce more complex geometries and patterns. However, the layer-by-layer material deposition used in the additive production process may leave visible ridges or staircase steps on the surface of the part, leading to unpredictable surface integrity, including higher roughness, hardness, and residual stress [5,6]. According to Wohlers’ 2023 report [7], postprocessing is one of the three main stages of the manufacturing process and accounts for approximately 27% of the additive manufacturing costs. In addition, various time-consuming and costly steps involved in postprocessing parts have been created through additive manufacturing. The key processes include the removal of the support material, finishing the surfaces, applying coloring and coatings, and conducting heat treatment. Each step can significantly affect the overall time and expenses associated with the final product preparation. Therefore, the machining of parts created through additive manufacturing is an essential post-production step required to attain a smooth surface texture suitable for part integration.

**Figure 1:**
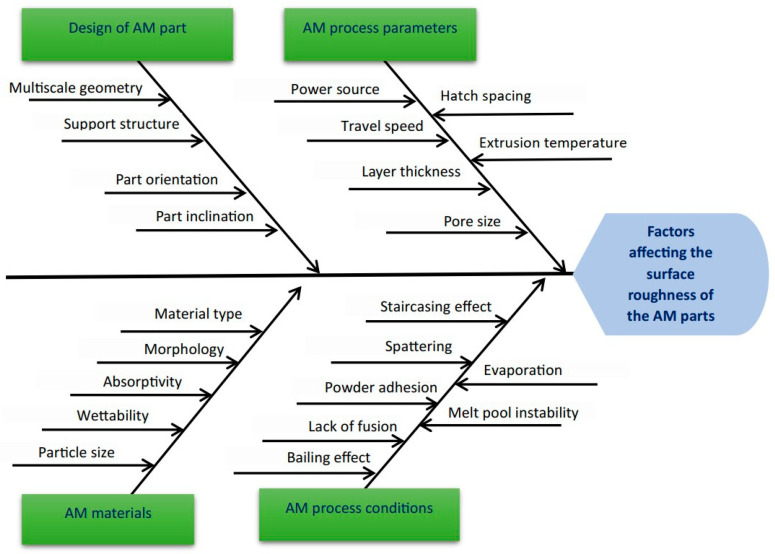
Fishbone diagram of factors affecting the surface quality of additively manufactured parts based on previous studies [1,5,15].

A set of techniques are used after the process to enhance the quality and functionality of the end product. An overview of the various factors affecting the surface quality of additively manufactured parts is presented in Figure 1, which uses a fishbone diagram to illustrate these influences. The surface finishing of additively manufactured parts can be categorized into chemical or mechanical methods. While mechanical finishing often involves abrasive machining techniques, it is not limited to them [8]. Common mechanical finishing methods for 3D-printed parts include vibratory bowl abrasion finishing, ultrasonic abrasion finishing, honing, polishing, lapping, milling, sandblasting, abrasive flow machining, and grinding [9,10]. Chemical finishing, on the other hand, employs non-abrasive processes such as chemical etching or electropolishing to achieve desired surface properties [11,12]. Surface-finishing techniques improve the surface marks and mechanical properties of additively produced parts. The abrasive finishing method has also been highlighted because of its ability to adequately finish additively manufactured parts with intricate geometries and hard-to-machine materials owing to its flexibility [9,13,14].

**Figure 2:**
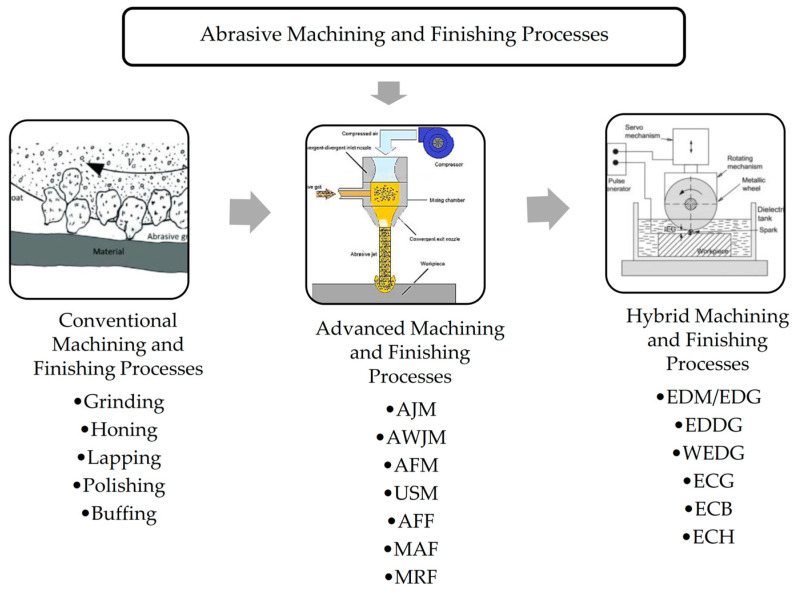
An overview of commonly employed abrasive machining and finishing processes based on references [16,20,21]. AJM—abrasive jet machining, AWJM—abrasive water jet machining, USM—ultrasonic machining, AFF—abrasive flow finishing, MRF—magnetorheological finishing, EDM/EDG—electrical discharge machining/grinding, EDDG—electrical discharge diamond grinding, WEDG—wire electrical discharge grinding, ECG—electrochemical grinding, ECB—electrochemical buffing, ECH—electrochemical honing.

Over the past two decades, abrasive machining and finishing have become crucial in manufacturing, particularly in creating effective and efficient production methods. Abrasive machining involves the removal of materials from a workpiece to achieve the desired shape and surface integrity for the intended function. This process uses numerous hard abrasive particles, either bonded or unbound, to remove materials through external mechanical forces [16]. Abrasive particles also play a significant role in various nano-finishing processes, including those utilizing loosely available abrasive particles mixed with magnetic particles [17]. Abrasive machining and finishing encompass a wide range of processes, from conventional methods such as grinding and honing to advanced techniques such as abrasive water jet machining and ultrasonic machining. These processes are further extended to micro- and nano-finishing techniques including abrasive flow machining and magnetic abrasive finishing. Each of these processes offers distinct advantages, and their application depends on the specific material being machined, the desired level of surface integrity, and the required accuracy [18,19]. Significant research has been conducted to utilize these processes for parts produced through additive manufacturing using metals, which will be examined in detail in the next section. Figure 2 illustrates the various machining and finishing processes that utilize abrasive particles to manufacture usable components. The figure offers concise schematic representations of these processes, along with depictions of traditional (grinding), advanced (AFM: abrasive flow machining), and hybrid (EDG: electrical discharge grinding) techniques used in abrasive machining [16,20,21].

This review aims to present recent research developments in abrasive machining and finishing of additively manufactured (AM) metal parts, exploring both conventional and nonconventional methods such as grinding, milling, abrasive flow machining, and electrochemical machining to assess their effectiveness in improving surface finish, dimensional accuracy, and mechanical properties. While several articles have addressed the mechanical and microstructural properties of metals fabricated via various AM methods, information on the machinability of specific alloys, including aluminum, titanium, cobalt, nickel, and steels, is limited. Understanding the influence of the inherent characteristics of different AM methods on the machinability of these alloys is critical, and this review provides a comprehensive overview of the AM processes for nickel, titanium, aluminum, cobalt, and copper alloys, along with an analysis of their machinability, while also identifying challenges and future trends in the machining of AM parts, emphasizing the need for further research into hybrid postprocessing techniques and the integration of AI for process optimization, ultimately contributing to the broader adoption of AM technologies across various industries.

## 2. Additive Manufacturing of Metals

AM represents an innovative manufacturing approach in which components are constructed through layer-by-layer addition of materials on the basis of 3D computer-aided design (CAD) models. Although it was initially developed for rapid prototyping, AM has gained traction across various domains, including product development and efficient production. Its popularity stems from its ability to create intricately shaped parts with minimal waste, which diverges from traditional subtractive manufacturing methods. By utilizing energy sources, such as lasers, electron beams, and electric arcs, materials are incrementally added to shape the desired object. Notably, polymers (51%) and metals (19.8%) are the predominant materials used in industrial 3D printing services [22].

Various materials can be used in AM processes. Among them, metal products have attracted the attention of scientists and businesses. Owing to the CAD models, the metal additive manufacturing process is used to create metals with greater creative freedom without wasting material. It uses powder or metal as the raw material and uses energy (such as a laser or laser injection, an electron beam, and binder jetting) to build the material layer by layer. Additive metal products can provide environmental benefits such as reducing waste, improving quality, reducing pollution emissions, and increasing the stability of the economy according to production needs [15,23]. All metal additive manufacturing (MAM) processes yield varying properties owing to their distinct techniques. These differences significantly affect the necessary postprocessing, particularly machining, for various applications. Therefore, it is essential to comprehend both the MAM processes and their application areas to formulate suitable machining strategies.

The American Society of Testing and Materials (ASTM) categorizes AM processes into seven groups, as outlined in ISO/ASTM 52900:2015 [24]. These methods include material extrusion, VAT photopolymerization, powder bed fusion (PBF), binder/material jetting, directed energy deposition (DED), and sheet lamination. Among these, only five are commonly used metals (Table 1). According to Wohlers’ study [7], most metal additive manufacturing systems utilize PBF processes, which accounted for 54% of the metal AM market in 2020. Metal extrusion technologies represent 10% of the market, whereas DED technology accounts for 16%. Additionally, material jetting (MJ) and binder jetting (BJ) collectively constituted the remaining 16% of the metal AM market during the same period. Table 1 provides an overview of the commonly employed MAM technologies within these categories, considering factors such as material type and form, resolution and accuracy, production speed and scale, process technology, object construction methods, and postprocessing requirements.

**Figure 3:**
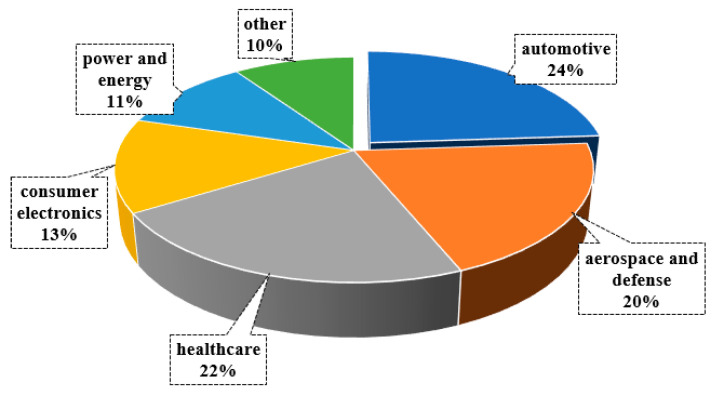
Literature survey on industrial applications of additive manufacturing in 2023, prepared by the authors on the basis of [7].

According to the global industrial metal 3D printing market [7], as shown in Figure 3, the automotive market segment will have a global revenue share of over 24% by 2023. The aerospace, defense, healthcare, and automotive sectors are expected to contribute to the growth of steel 3D printing during the forecast period because of the tight use of technology in many manufacturing processes associated with vertical building.

## 3. Machining of Additively Manufactured Metals

### 3.1. Conventional Methods

#### 3.1.1. Grinding

**Figure 4:**
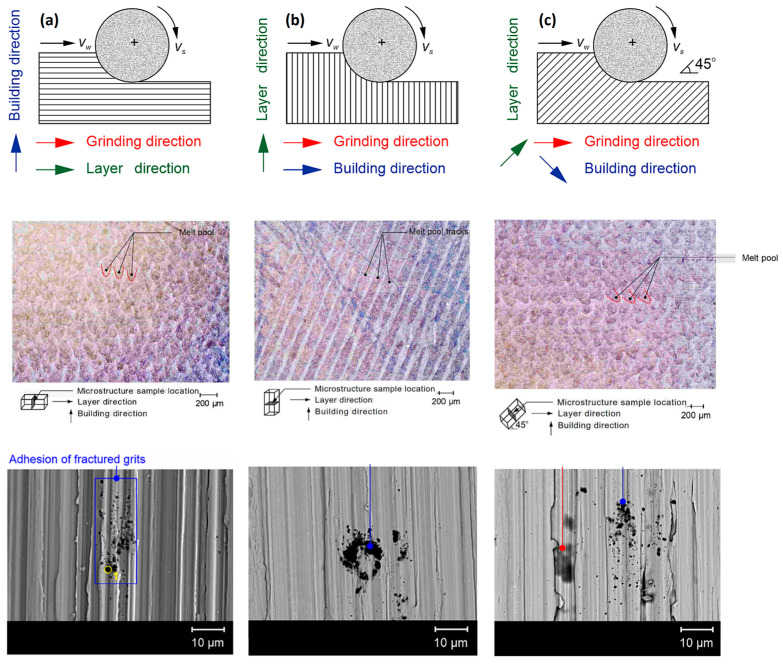
SEM images and EDS analysis illustrating the adhesion of abrasive particles and the occurrence of burn marks on the ground surface of as-built SLM-printed Inconel 718 [25], with grinding directions (**a**) along the layers, (**b**) perpendicular to the layers, and (**c**) 45 degrees to the layers. SEM—scanning electron microscopy, EDS—energy-dispersive spectroscopy.

Grinding is among the most widely applied conventional abrasive tools used to obtain a smooth surface finish and achieve a highly precise dimension size. However, it may not completely eliminate the surface concerns implied during additive manufacturing. Furthermore, a research survey revealed that grinding can be used as a finishing postprocessing technique for additively manufactured metal materials. Among these, Inconel 718 (a superalloy of nickel), Ti6Al4V, Ti-5553 (titanium alloy), CoCrMo (cobalt alloy), and AISI 316L (aluminum alloy) are commonly grindable additively manufactured metals. In a recent investigation, Vaisakh and Dinesh [25] analyzed the machinability of SLM-printed Inconel 718. The primary concern of this research was the determination of the specific grinding forces and grindability of selective laser melting-printed Inconel 718 compared with those of wrought Inconel 718. To determine whether SLM-printed Inconel 718 is easy to grind and to establish the appropriate forces, the author analyzed the specific forces required for grinding. The specific forces were obtained by considering the number of passes and the depth of cut. Grinding SLM-printed Inconel 718 has advantages over traditional wrought Inconel 718. This is because the micro-grit fracturing observed in SLM-printed Inconel 718 reduces the impact of strain hardening on the specific energy, even though the hardness increases compared with that of wrought Inconel 718. On the other hand, SEM experiments were conducted on the as-built Inconel 718 for three grinding directions, as shown in Figure 4: along the layer (a), perpendicular to the layer (b), and 45° to the layer (c). All directions presented similar challenges owing to the inherent porosity and brittle fracture of the abrasive grits. Grinding has several benefits over other machining processes, i.e., milling, including achieving the desired tolerance levels and featuring compressive residual stress [26]. Similarly, Chadha et al. [27] presented the advantage of grinding PBF-printed metals, particularly in terms of reducing the residual stresses. According to the aforementioned studies, the porous structure of additively manufactured samples intensifies the brittle fracture of abrasive grits during the grinding process, resulting in an inferior surface quality even though it reduces the strain-hardening effect. This results in further postprocessing techniques, such as heat treatment, which are significant for the reducing porosity and pore size [28]. Some studies have indicated that the use of targeted cooling fluids during the grinding of additively manufactured titanium alloy components is preferable [29]. Grinding with targeted cooling fluids has several advantages, such as enhancing the surface quality and decreasing the tensile residual stress. Unlike conventional surface grinding, smaller workpieces and more profound cuts are required to grind additively manufactured components. As a result, Kirsch et al. [30] employed a creep feed grinding mode based on selective laser melting. Moreover, an innovative weakened porous material can be utilized as a support structure to improve the grindability of additively manufactured elements [31]. With the aid of the grinding process, the surface quality increased significantly. Cryogenic grinding allows improved surface characteristics of additively produced Ti-6Al-4V to be obtained, resulting in the best surface finish and reducing the initial roughness (Ra) from 5.94 µm to 0.259 µm [32]. Moreover, the grinding forces were 57% lower than those of the conventional processing methods. The surface roughness with dry grinding was 0.356 µm, with elevated temperatures causing an increase in the microhardness.

#### 3.1.2. Milling

**Figure 5:**
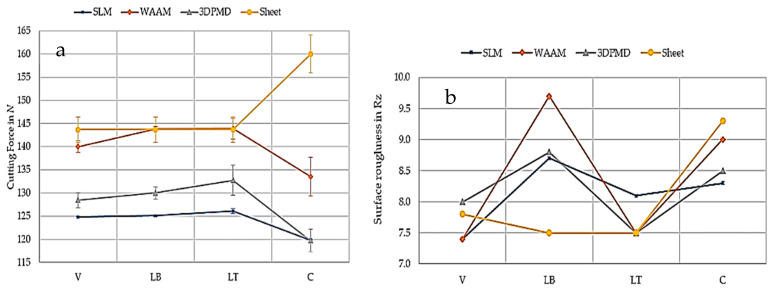
Machining properties of samples manufactured via additive manufacturing processes [33]. (**a**) Cutting force. (**b**) Surface roughness. V—velocity, LT—layer thickness, C—cooling rate, LB—laser beam.

Another useful and successful conventional postprocessing method for finishing additively manufactured metals is milling. Research has shown that the additive manufacturing process leads to notable variations in machinability, indicating that the mechanical properties alone are insufficient for full characterization. For example, Laue et al. [33] reported that in milling the properties of parts manufactured via additive manufacturing technologies mainly rely on manufacturing processes such as milling force, temperature, and surface roughness, which is also supported by Fei et al. [34]. As shown in Figure 5a, the SLM samples presented the lowest overall cutting forces, with minimal variation across different directions (vertical, layer build, and layer thickness). A similar decision was made by other published works [35]. In contrast, the 3DPMD samples had slightly greater cutting forces (approximately 5 N greater than that of SLM). The milling forces varied with the direction, particularly when the vertical-to-layer build and layer thicknesses were compared, which revealed greater forces against the feed direction. The WAAM samples showed no difference in the milling force along the wall but exhibited small differences lengthwise and vertically, along with significant variations across the wall. In the other results of the above research, as shown in Figure 5b, the differences in surface roughness among the various manufacturing processes and removal directions were minimal, with values around Rz = 10 µm, indicating a transition between standard and fine manufacturing accuracy. The variation in roughness was limited to a maximum of 2 µm, suggesting that AM had no significant effect on the surface roughness. However, a contradictory decision was made by some authors in another work [36,37,38,39], in which the surface roughness decreased with increasing cutting force while milling parts were produced by WAAM. This demonstrates the necessity for an increased focus on postprocessing machining operations following WAAM to develop optimal strategies that reduce tool wear while ensuring high surface quality and production rates. Generally, the finishing of additively manufactured parts, such as HSLA steel, Ti-6Al-4V, AlSi10Mg, AISI 316L, IN718 alloy, 316L stainless steel, and GH4169 alloy, relies on conventional and climb milling methods [40,41,42,43].

#### 3.1.3. Polishing

Mechanical polishing uses fine abrasive particles to produce a specific surface texture. These particles are typically combined with a medium to form a slurry, which is then applied to the workpiece surface by using a cloth pad to gradually achieve a smoother finish. This process is controlled by the applied force. A study by McGaffey et al. [44] examined postoperative surgical site infections (SSIs) related to metal implants and reported that manual polishing of metal 3D-printed implants significantly reduced biofilm formation on the implant surface compared with untreated surfaces. The materials analyzed in this study included Ti6Al4V, CoCr, and 316L stainless steel. Karakurt et al. [45] introduced a new technique for polishing 3D-printed enclosed structures with magnetic–abrasive slurries. The method employs four types of slurries containing silicon carbide and alumina abrasive particles of varying sizes. Initial findings indicate significant enhancements in surface roughness for copper structures, decreasing it from 35 µm to approximately 4 µm. This demonstrates the process’s effectiveness in polishing additively manufactured copper components. Several methods can be used to enhance the outer surfaces of L-PBF components, including blasting and laser polishing. However, the demand for and complexity of polishing the inner surfaces of additively manufactured components are much greater. In recent years, researchers have made progress in developing different polishing techniques specifically designed for the internal structures of 3D-printed parts, such as L-PBF components. These methods can be classified into four categories on the basis of their mechanisms: mechanical, chemical, electrical, and hybrid [46,47]. Notably, all the aforementioned methods have benefits and drawbacks in their use for L-PBF internal structures. Because the number of surface features in parts fabricated with the help of L-PBF is too high, it is essential to research and establish a link between the features and phenomena of material removed during the polishing process. Internal structures cause problems in the design and deployment of the necessary equipment. As a result, it is necessary to take significant steps to create exceptional polishing machines that improve the productivity and ensure the final surface quality of L-PBF internal structures. Compared with fabrication optimization and hybrid processing techniques, surface polishing methods that involve material removal are generally more cost-effective for eliminating residual powders and achieving a smoother sintered surface [48].

#### 3.1.4. Lapping

Lapping is a widely utilized technique for achieving superior surface finishes, minimal subsurface damage, and high dimensional and shape accuracy, particularly in materials like metals, ceramics, and hard, brittle substances with porous structures [49]. It effectively addresses waviness and subsurface defects from prior processes. Despite its long history, lapping remains a focus of ongoing research. Modern advancements include methods such as slurry-free or free abrasive lapping, where abrasives are directly applied to the lapping wheel, similar to grinding [50]. Lapping enhances the adhesion of physical vapor deposition coatings, such as CrAlN, on 316L stainless steel substrates produced using laser powder bed fusion technology [51]. Adhesion can be achieved mechanically through an even treatment of the processed material. Lapping processes were also employed to successfully polish the surface pointing the sensor array, preventing punctures in the subsequent insulating polyamide layer while ensuring the material’s optimal properties for the intended application [52]. In their review papers, Koneru et al. [53] and Bhatt et al. [54] concluded that lapping can be applied as a finishing technique in situ for both magnetic and non-magnetic materials, such as superalloys, ceramics, and biomaterials. The selection of machine tools and attachments for lapping is determined by the surface characteristics, while the surface quality obtained, material removal rate, and the flexibility of the magnetic abrasive brush are affected by the suitable selection of process parameters.

#### 3.1.5. Honing

Honing is an abrasive process in which bonded grains are used to finish rough, semi-finished, or finished cylindrical holes. In the field of precision machining, honing plays a crucial role in the production of high-precision functional components on the basis of current technological standards. Typically, this process is the final machining step in the manufacturing series and meets strict requirements for shape, dimensional accuracy, and surface quality, achieving tolerances of less than 1 μm [55,56]. This involves the removal of material through friction between the abrasive tool and the surface of the part [57]. For example, honing machining has been used to optimize the process dynamics of internal long-stroke honing by reducing the mass of the workpiece fixture using topology optimization and SLM [58]. Thus, the SLM-manufactured mass-reduced workpiece fixture exhibited significantly greater oscillation amplitudes at higher rotation speeds than did the conventional fixture. Although the allowable factors of honing machining include an improved surface finish and tensile accuracy and the ability to address bubbles in internally manufactured projects, their current utilization degree is restricted by actual limitations. These include the quantity of material that can be withdrawn during a time-consuming and intricate computational process, together with the connected price levels. Consequently, more extensive identification is needed to fully evaluate uprightness and determine when it is practicable [59,60].

#### 3.1.6. Sandblasting

For materials produced through the additive manufacturing process, sandblasting is frequently employed in a non-destructive manner to augment external surfaces, especially during deburring [61]. Sandblasting is a widely used mechanical abrasion-cleaning technique that is highly effective in enhancing the surface texture of parts produced by SLM. Bernevig-Sava et al. [62] employed sandblast machining techniques following SLM processing, which specifically targeted external surfaces. The samples were subjected to single sandblasting, two consecutive sandblasting, or no sandblasting. The roughness (Ra) of the outer surfaces was evaluated for all the samples, indicating a reduction in the height of the micro-irregularities and a more uniform roughness profile as the sandblasting intensity increased. Studies have also focused on optimizing sandblasting process parameters to address the increasing demand for scaffold applications. For example, Yu et al. [63] investigated SLM-printed Ti6Al4V intervertebral cage samples treated with sandblasting, leading to the identification of an optimized sandblasting process. The treatment utilized alumina sand grains that were used to enhance the surface quality of the intervertebral cages. Similarly, the sandblasting process parameters were optimized for finishing the 3D-printed high-frequency apparatus used in communication systems [64]. The impact of several postprocessing techniques, including sandblasting, electrolytic polishing, chemical polishing, and abrasive flow polishing, on the surface quality of Ti6Al4V parts created with LPBF was investigated by Lu et al. [65]. The authors reported that sandblasting is an effective processing technique that can reduce surface roughness by 70%. Sandblasting involves propelling grit with sharp edges onto parts at high speed through airflow. This method resulted in the lowest surface roughness; however, it is also important to mention that the surface was contaminated with brown corundum residues from sandblasting.

### 3.2. Nonconventional Methods

Nonconventional or nontraditional abrasive machining techniques offer promising alternatives to address the limitations faced by conventional methods in achieving superior finishes on additive-manufactured parts without the need for sharp cutting edges. The unique characteristics of additive manufacturing, such as intricate geometries, internal channels, and varying material properties, pose challenges to conventional techniques. In response, nonconventional abrasive machining techniques, such as abrasive flow machining, electrochemical machining, magnetic abrasive finishing, and vibratory bowl finishing, have emerged as viable solutions to overcome these limitations and enhance the surface quality of additively manufactured metal parts.

#### 3.2.1. Abrasive Flow Machining

**Figure 6:**
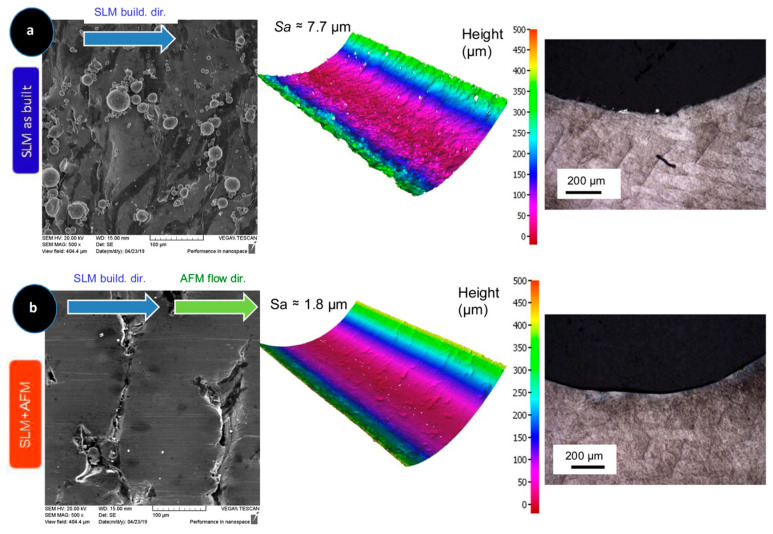
Surface topographies of (**a**) SLM as-built samples and (**b**) SLM + AFM [69].

AFM, a technique specifically developed for internal surface finishing, offers a novel approach to achieve exceptional precision on internal surfaces, and is capable of attaining a roughness of (Ra) = 0.2 µm or lower [66]. Initially, Kim pioneered the use of this method for internal deburring [67]. Yin et al. [68] subsequently applied AFM to polish the microchannels of mechanical components. Recently, according to Han et al. [69] the use of abrasive flow machining in SLM-produced channels has been effective in achieving a significant improvement in mold surface roughness and an increase in fatigue life. According to the aforementioned work, the SLM as-built channels, as shown in Figure 6a, display local waviness owing to the layer-by-layer deposition process, along with visible partially melted and unmelted powder grains. The optical micrographs revealed irregular and rough surface patterns. In contrast, Figure 6b illustrates that AFM polishing effectively removed many of these irregularities, although some microvalleys remained, indicating that AFM primarily eliminated the higher peaks. As a result, the surface roughness measurements confirmed that the SLM as-built samples had a roughness of 7.7 μm, whereas the SLM + AFM samples exhibited a significantly smoother surface with a roughness of 1.8 μm, which is in agreement with the result of the study conducted by Jia et al. [70]. Additionally, using hydrogel-based abrasive media, AFM has been utilized to achieve effective finishing of SLS-printed femoral heads [71]. Shaik et al. [72] investigated the use of a natural polymer-based abrasive medium in abrasive flow finishing (AFF) to significantly reduce surface roughness in Atomic Diffusion Additive Manufacturing (ADAM) copper parts, highlighting the effectiveness of various process parameters. The results showed reductions of up to 88.15% in surface roughness after optimal processing conditions. Zhang et al. [73] applied the AFM method to decrease the surface roughness of additively manufactured parts made of titanium with thin walls and internal channels. The results of this study led to the conclusion that AFM provides a significant reduction in the average value of the surface roughness and its standard deviation in the case of larger internal channels. However, their limited accessibility limits their ability to reach and eventually complete the internal channels and hidden surfaces. Consequently, this leads to incomplete or irregularly finished internal features. Consequently, their overall functioning and performance are negatively affected [74,75,76,77].

#### 3.2.2. Electrochemical Machining

**Figure 7:**
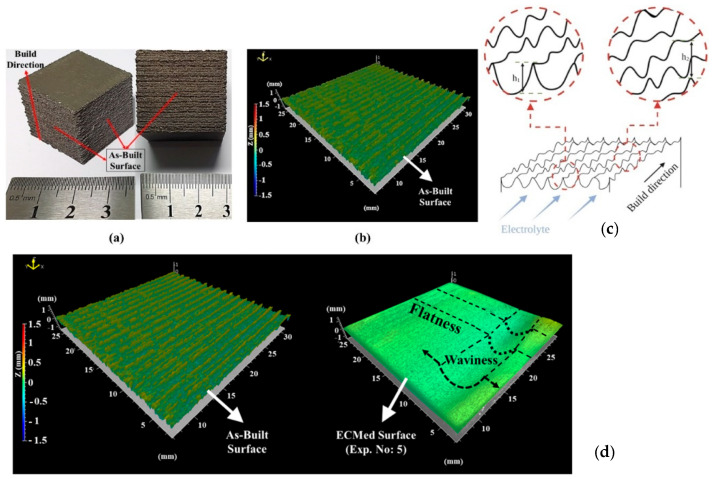
Surface morphology of the EBM-printed γ-TiAl alloy samples [80]. (**a**) As-built surface build direction; (**b**) surface topography result; (**c**) change in peak geometry; (**d**) surface morphologies before and after ECM.

Electrochemical machining (ECM) is considered a promising advanced machining technique for precise and accurate machining of conductive hard metals. Because electrochemical polishing has several benefits, including no tool wear, no effect on the surface hardness, and minimal mechanical force, it can be used to polish the interior surfaces of curved structures that are difficult to machine [78]. In a recent study, Demirtas et al. [79] demonstrated that electrochemical machining (ECM) could successfully reduce the surface roughness of additively manufactured γ-TiAl parts. According to this study, the feed rate and electrolytic conductivity are the main parameters of electrochemical machining that affect the surface quality of additively manufactured γ-TiAl parts. Consequently, reductions in surface roughness of 98.6% and 98.5% were observed for Sa and Sq, respectively. The authors also reported that the surface roughness significantly decreases to 0.96 for Sa and 1.29 for Sq when the ECM parameters are set to a feed rate of 1 mm × min^−1^ and an electrical conductivity of 125 mS × cm^−1^. However, despite achieving a low Sa value, the surface waviness and prevalence of the peaks were noticeable (see Figure 7a,b). These peaks are attributed primarily to the melted particles on the surfaces of the electron beam melting (EBM) parts (Figure 7c,d). Low roughness values vary based on conductivity levels, which are influenced by the cathode material and electrical conductivity of the electrolyte [80]. According to Khan et al. [81], surface roughness initially increases with increasing electrolyte conductivity. However, beyond a certain conductivity threshold, further increases can adversely affect surface integrity. Additionally, Kim and Park [82] studied the use of electrochemical polishing (ECP) to enhance the surface quality of 3D-printed metal parts made through SLM with STS316L material. They reported that ECP significantly improved the surface quality by reducing the surface roughness by up to 75% and eliminating irregularities, rough peaks and valleys, and unnecessary particles. Furthermore, ECP enhances the brightness and reflection of the surface. Lynch et al. [83] proposed and demonstrated a method for enhancing the surface finish of metal lattices in additive manufacturing by utilizing the COOLPULSE ECM process. This study specifically examined Inconel 718 lattice coupons produced via LPBF. The findings indicate that the proposed approach successfully eliminates material from internal surfaces, leading to enhanced roughness across the entire lattice framework, in contrast to methods that focus solely on external blasting. In another study, surface finishing of an SS316L part was performed by L-PBF using toolless electrochemical jet machining (ECJM), and investigations were performed to evaluate the different aspects of surface integrity [84]. The experimental findings showed that ECJM eliminated the surface imperfections and irregularities that were highlighted in the as-deposited samples. After postprocessing, the mean surface roughness improved by 72%. Consequently, the study concluded that the special feature of the toolless electrochemical machining process is appropriate for finishing AM parts. Thus, recent advancements in electrochemical polishing technology have opened new avenues for machining previously unattainable materials and shapes.

#### 3.2.3. Magnetic Abrasive Finishing

The MAF process is a special and efficient technique used to finish both the internal and external surfaces of workpieces. This is particularly useful for achieving precise finishes for a wide range of items, including biomedical devices and automotive components such as shafts and crankshafts [85,86]. Zhu et al. [87] employed magnetic abrasive finishing (MAF) to polish the surface of 316L stainless steel produced through SLM at various building angles. They observed a remarkable improvement in surface roughness initially from 4–10 μm to approximately 0.1 μm in addition to successfully eliminating surface defects such as unmelted particles and balling effects. However, they observed that the optimal parameters for the MAF remained consistent for the same material across different forming angles, although the polishing time required varied significantly. Similarly, experimental findings have confirmed that MAF has a limited capacity to remove support structures during SLM processing. In another study, Zhang and Wang [88] introduced magnetically driven internal finishing (MDIF) to enhance the surface quality of AISI 316L stainless steel tubes produced through SLM. Through single-point polishing experiments, this study revealed that the surface roughness significantly improved from an initial value of 11.599 μm Ra to 0.385 μm Ra. In the section-polishing experiments, the surface roughness reached Ra = 0.808 μm. In addition, a comparative analysis was performed between MDIF and conventional MAF. The findings indicated that MDIF surpassed MAF in terms of the final surface finish and efficiency. The authors attributed this observation to the trapping of abrasive particles on the rough surface of the workpiece combined with the application of a minimal finishing force. In a study conducted by Cui et al. [89], the surface quality of AlSi10Mg alloy curved surface samples was examined using MAF for machining parts produced through SLM. The objective of this investigation was to enhance the surface quality of the AlSi10Mg parts. The results demonstrated that the implementation of the 75° trapezoidal-slotted permanent MAF tool significantly reduced the surface roughness to 0.279 μm. Moreover, magnetic finishing technology increased the surface roughness of the AlSi10Mg alloy SLM-formed samples. Although the surface hardness of the samples remained unchanged, a significant improvement in the hydrophobicity of the surface was observed.

#### 3.2.4. Vibratory Bowl Finishing

**Figure 8:**
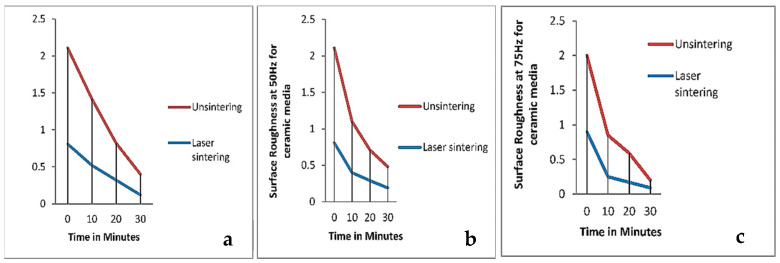
Surface roughness of the laser-sintered and unsintered Inconel 718 samples at (**a**) 25 Hz, (**b**) 50 Hz, and (**c**) 75 Hz frequency and 0.8 mm amplitude [90].

Vibratory bowl finishing is a commonly used mass-finishing process for mass production in several industries. The workpiece was placed in a bowl or tube filled with abrasive media and supported by springs as it was subjected to a shaking motion using vibrators connected to a revolving spindle [90]. The shaking results in the creation of normal and hydrostatic forces as a result of the weight of the media and impact forces. An advantage of vibrating is that it can be fully automated, and it is better at handling the parts. Vibratory bowl finishing is a feasible postprocessing technique for AM parts [91,92,93]. These approaches are characterized by the rapidity of time constructions and postprocesses, which can be automated for serial production. For example, Kil et al. [94] investigated a vibro-finishing process applied to AlSi10Mg samples produced using L-PBF. The results demonstrated a substantial reduction in the surface roughness, which decreased from 44 µm to 4.25 µm. This improvement occurs progressively, as the most exposed peaks are leveled, resulting in an isotropic surface. The study also noted that the abrasive nature of vibro-finishing leads to slight rounding of sharp edges, which is an important consideration. Parts with thin edge details should not undergo this finishing process, because they may become delaminated and develop burrs. Additionally, large-mesh sections are unlikely to withstand vibratory finishing. This decision was also consolidated in the chapter of a book published in work [95]. On the other hand, Karthik et al. [90] clearly described vibratory surface finishing (VSF), which is characterized by frequency and amplitude (the distance traveled by the medium owing to vibratory motion) at various times, for the application of SLS-manufactured Inconel 718 samples. The results, as illustrated in Figure 8, show that laser-sintered samples exhibit a lower rate of surface roughness at a frequency of 75 Hz than unsintered samples do at a similar frequency. This improvement is attributed to the productive impact of the ceramic media on the samples at 75 Hz as well as the perpendicular orientation of the components. Nezarati et al. [96] presented one of the first academic studies on the surface integrity and dimensional deviation of SLM-produced SS316L specimens after VSF. After 9 h of VSF, SLM-Ra decreased by 75%, from 6.72 to a minimum of 1.68 μm with a spherical media. According to [97], the vibratory mass-finishing technique effectively removes peaks from the surface of selective laser melted GRCop84 copper alloy but does not eliminate valleys, resulting in a profile that resembles an extruded waveguide. This occurs because vibratory finishing primarily targets the high points on the surface, leading to a smoother overall finish while leaving deeper valleys intact. These valleys may still trap contaminants or contribute to radio frequency (RF) losses, which can initiate arcing if not adequately addressed. The unevenness is likely due to the method’s limitations in fully addressing the intricate surface topography created during the selective laser melting process.

## 4. Discussion

Advances in additive manufacturing have revolutionized industry, and the creation of complex geometries and intricate internal structures has become more feasible than traditional subtractive manufacturing methods. This unique layer-by-layer build-up associated with each AM process is often prone to surface quality problems such as apparent ridges, staircase effects, and unpredictable surface integrity. Most importantly, machining, particularly abrasive machining and finishing, has played a key role in overcoming these surface quality problems and qualifying AM-manufactured parts for practical applications. In this regard, well-established and nonconventional machining techniques investigated thus far for postprocessing operations on AM metal parts have been discussed in the literature review. To date, traditional machining techniques, such as grinding, milling, polishing, honing, and sandblasting, have been widely surveyed for their ability to improve the surface finish, dimensional accuracy, and mechanical properties of AM metal components. This review shows that the inherent features of different AM processes, such as porosity, microstructural features, and residual stresses, may significantly affect the machinability of the produced parts. For example, during the grinding of SLM-printed Inconel 718, although less severe strain-hardening effects can be produced, the porous structure and brittle fracture behavior of abrasive grits are predisposed to yield poor surface quality. In addition, studies have been conducted on nonconventional abrasive machining techniques, such as abrasive flow machining, electrochemical machining, magnetic abrasive finishing, and vibratory bowl finishing. These advanced methods have the potential to solve the difficulties in machining internal geometries with complicated forms and hard-to-machine material combinations that are often encountered in AM parts.

Overall, the most commonly used unconventional machining methods for the surface machining of additively manufactured parts are discussed. However, several methods, such as abrasive water jet machining, electrical discharge machining, ultrasonic machining, laser processing, laser-assisted machining, and friction stir processing, need further investigation for use in machining of additive manufacturing parts. Each method has specific contributions and limitations. The key challenges are the possibility of thermal and chemical damage, limited work on polishing metal parts with deep inner holes, and the possible risk of thin-walled structural damage owing to excessive pumping pressure or abrasive contamination of internal channels. However, based on the type of abrasive tool, in combination with other factors, the mechanical properties of fracture toughness and ductility, fatigue strength, surface quality, dimensional accuracy, and material behavior can be enhanced. A choice should be made based on final requirements, material properties, desired outcomes, and other factors.

Table 2 summarizes the most recent research on the average reduction in surface roughness achieved using the most commonly employed abrasive tools for finishing additively manufactured metals, particularly those produced by LPBF.

**Figure 9:**
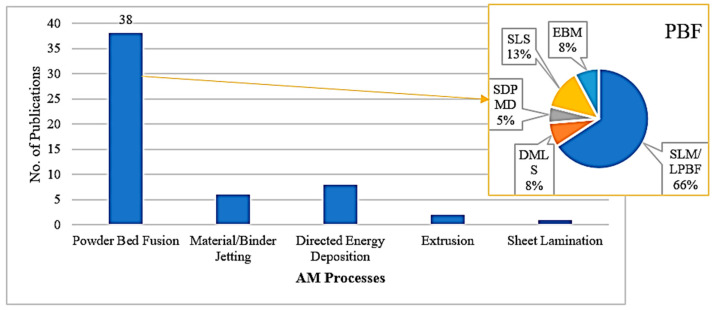
A literature survey on the machining of additive manufacturing technologies.

**Figure 10:**
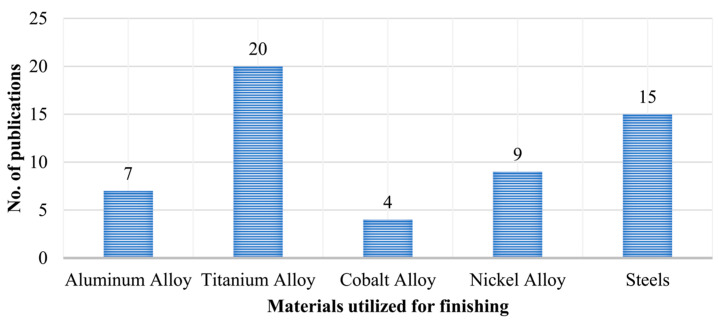
A literature survey on the machining of additively manufactured metals.

In this study, the machining characteristics of additively manufactured metal components were reviewed extensively. In total, 55 original studies on machining were considered in this review. Figure 9 presents publication trends across various machining processes of additive manufacturing from the period of 2020-to-2024, with powder bed fusion being the most extensively researched (38 publications), in which the SLM/LPBF technique accounted for approximately 65% of the papers. In contrast, extrusion and sheet lamination have received relatively less research attention, with five and three publications, respectively, suggesting the need for further exploration of AM techniques. Additionally, as seen in Figure 10, the titanium alloy is the leading material utilized for finishing in 37% of publications, followed by steel in 28%, nickel alloy in 17%, aluminum alloy in 13%, and cobalt alloy, with the lowest percentage, in 5% of publications. This reflects the significant interest in utilizing titanium and steel alloys, likely because of their desirable properties and widespread applications in the aerospace, automotive, and machinery industries, where optimized machining and finishing techniques are crucial for enhancing the performance of additively manufactured parts.

This systematic review highlighted several challenges, which are listed as follows:

⮚ The inherent porosity and high number of surface features of additively manufactured metals make effective machining difficult.
⮚ In some cases, the brittle fracture of abrasive grits during machining leads to an inferior surface quality, despite the reduced strain-hardening effects. This leads to additional postprocessing (e.g., heat treatment).
⮚ During the application of unconventional machining methods for the surface finishing of additively manufactured parts, there is the possibility of thermal or chemical damage as well as the limited effectiveness of polishing metal parts with deep internal holes.
⮚ The risk of thin-walled structural damage due to excessive pumping pressure or abrasive contamination of internal channels poses a significant challenge for the use of unconventional machining methods on additively manufactured components.
⮚ Metal additive manufacturing processes, such as laser metal deposition, can result in rough surfaces, discoloration, and unmelted particles owing to the repeated instant dissolution and solidification of the metal. These challenges make it difficult to achieve high-quality surface finishing, especially for direct application in the food and biomedical industries.

Future Trends:

❖ The future studies should focus on achieving an optimal balance between porosity and strength in the additive manufacturing of machining tools.
❖ Further studies on the combination of various postprocessing techniques for optimizing the surface finish and structural integrity of AM parts are required for synergetic hybridization.
❖ The demand and complexity of finishing the inner surfaces of LPBF components are greater compared to outer surfaces. However, there is a lack of comprehensive research on the overall effects of polishing on internal structures. Therefore, future research could focus on the efficient inner-surface-finishing methods.
❖ In addition, studies related to the selected AM methods and the machinability of certain new alloy compositions could be helpful. This will help gain better insight into the positioning of AM in various manufacturing industries, such as automotive and aerospace.
❖ Furthermore, studies could investigate the incorporation of machine learning or artificial intelligence algorithms to optimize machining parameters, improve surface quality, and reduce flaws.
❖ The inherent porosity of additively manufactured metals complicates tools, especially the grinding process. Therefore, future research could focus on additively manufactured machining tools and their specific applications.

## 5. Conclusions

This review highlights the growing importance of abrasive machining and finishing techniques for improving the surface quality and dimensional accuracy of additively manufactured parts. This review demonstrates that both traditional and nontraditional machining processes, including grinding, milling, abrasive flow machining (AFM), and electrochemical machining (ECM), have distinct advantages and limitations. Traditional methods are still applicable in less complex postprocessing operations, especially those involving external surfaces. However, more sophisticated techniques, such as ECM and AFM, achieve accuracy on internal channels and complex geometries with substantial reductions in surface irregularities. These nontraditional methods are capable of reducing roughness without sacrificing the structural integrity of complex internal surfaces, which has become increasingly important in a range of high-precision fields.

The optimization of these machining processes for specific AM materials and hybrid approaches, in which several techniques are combined to improve quality and efficiency, should become the focus of future research. Another aspect that will be of great importance is the effects of these postprocesses on mechanical properties, including fatigue life and corrosion resistance when AM parts are used in load-bearing applications. The industry can further tap the potential that lies in the advancement of knowledge concerning machining techniques and their interaction with AM materials to further improve the economic viability and performance of additively manufactured components, thus driving wider adoption across sectors. In this study, while challenges still exist in the machining of AM parts, ongoing developments in finishing techniques provide significant opportunities for extending the applications of AM technologies.

## Figures and Tables

**Table 1:** Overview of commonly employed MAM categories based on some AM process categories outlined by ISO/ASTM 52900:2015 and references [10,23].

Criteria	Powder Bed Fusion	Material/Binder Jetting	Extrusion	Directed Energy Deposition	Sheet Lamination
Material Form	Powder	Liquid resin	Plastic-bound metal powder	Wire or powder	Paper
Process Technology	SLS, SLM, DMLS, LPBF, EBM, MJF, SHS	PJM, MJM, NPJ, DOD	ADAM, CEM, FDM, BMD, MIM	WAAM, EBAM, LMD	LOM, UAM, SDL
Resolution and Accuracy	High	High	Moderate-to-high	Moderate-to-high	Moderate
Speed and Production Scale	Moderate-to-slow; small-to-medium-scale.	Fast; large-scale.	Moderate-to-fast; medium-to-large-scale.	Moderate; medium-scale.	Low-to-medium; prototyping and low-volume.
Way of Building the Object	Melting or sintering powdered material layer by layer.	Print heads jet liquid binding agents onto a layer of metal powder.	A metal filament is heated and pushed through the print head’s nozzle.	Depositing and fusing metallic powders or wires/rods layer by layer using a focused energy source.	A laser or blade crops thin sheets of metal, layer by layer.
Postprocessing Requirements	Machining, surface finishing, and heat treatment.	Machining, surface finishing, and may require infiltration or sintering.	Debinding, sintering, machining, and coating often required.	Machining, surface finishing, and may require heat treatment.	May be required for strengthening and surface finishing.

SLS—selective laser sintering, DMLS—direct metal laser sintering, MJF—Multi Jet Fusion, SHS—selective heat sintering, PJM—PolyJet Matrix, MJM—Multi Jet Modeling, NPJ—nanoparticle jetting, DOD—drop on demand, ADAM—atomic diffusion AM, FDM—fused deposition modeling, BMD—bound metal deposition, MIM—metal injection molding, WAAM—wire arc additive manufacturing, EBAM—electron beam AM, LMD—laser metal deposition, LOM—laminated object manufacturing, UAM—ultrasonic AM, SDL—selective deposition lamination.

**Table 2:** Summary of past research on abrasive tool surface quality improvement methods.

Abrasive Tools	AM Techniques	Material Utilized and Features	Avg. Reduction in Surface Roughness, in %	Ref.
Grinding	L-PBF	Inconel 718 AISI 316L Ti-6Al-4V	94	[25,28,32]
Milling	L-PBF	AlSi10Mg, AISI316L, IN718, 316L, GH4169, Ti-6Al-4V	98.3	[35,40,42]
Polishing	L-PBF	18Ni, AlSi7Mg	80	[46,48]
Lapping	LPBF	GCr15, 316 L	83.89	[51,53]
Honing	L-PBF	Ti-6Al-4V	83.34	[58]
Sandblasting	L-PBF	Ti6Al4V, Co-Cr alloy	70	[61,65]
Abrasive Flow Machining	L-PBF	Ti-6Al-4V	76.6	[69]
Electrochemical Machining	L-PBF	γ-TiAl, STS316L, SS316L	81.5	[82,84]
Magnetic Abrasive Finishing	L-PBF	316L AlSi10Mg	84.37	[87,88]
Vibratory Bowl Finishing	L-PBF	AlSi10Mg SS316L	83	[94,96]

## Data Availability

No new data were created or analyzed in this study. Data sharing is not applicable to this article.
